# Micropercutaneous nephrolithotomy versus retrograde intrarenal surgery in the treatment of renal stones: A systematic review and meta-analysis

**DOI:** 10.1371/journal.pone.0206048

**Published:** 2018-10-19

**Authors:** Xiaohang Li, Jiuzhi Li, Wei Zhu, Xiaolu Duan, Zhijian Zhao, Tuo Deng, Haifeng Duan, Guohua Zeng

**Affiliations:** 1 Department of Urology and Guangdong Key Laboratory of Urology, The First Affiliated Hospital of Guangzhou Medical University, Guangzhou, China; 2 Department of Urology, The People’s Hospital of Xinjiang Uygur Autonomous Region, Urumqi, China; University of Pennsylvania Perelman School of Medicine, UNITED STATES

## Abstract

**Objective:**

To compare the efficacy and safety of micropercutaneous nephrolithotomy (Microperc) and retrograde intrarenal surgery (RIRS) in treating renal stones using published literature.

**Methods:**

A systematic literature review was performed on August 21, 2017, using PubMed, Embase, and Cochrane Library databases in accordance with the PRISMA guidelines. Summarized mean differences (MDs) or odds ratios (ORs) with 95% confidence intervals (CIs) were used to assess the differences in outcomes between Microperc and RIRS.

**Results:**

A total of nine studies (7 in adult patients and 2 in pediatric patients) containing 842 patients (381 Microperc cases and 461 RIRS cases) with renal stones were included in this analysis. Among the adult patients, Microperc was associated with higher stone-free rate(SFR)(OR: 1.6; 95% CI, 1.03 to 2.48), significantly longer hospital stays (MD: 0.66 day; 95% CI, 0.17 to 1.15), longer fluoroscopy time (MD: 78.12 s; 95% CI, 66.08 to 90.15), and larger decreases in hemoglobin (MD: 0.59 g/dl; 95% CI, 0.16 to 1.02) than was RIRS. No significant differences were observed with respect to operative time, stone-free rate, complication rate or auxiliary procedures.

**Conclusions:**

Our results demonstrated that Microperc might be more effective in adult patients than RIRS will due to its higher SFR. However, longer hospital stays, longer fluoroscopy time and a larger decrease in hemoglobin should be considered cautiously.

## Introduction

Over the past two decades, open surgery has been almost completely replaced by minimally invasive procedures for patients with kidney stones (e.g., PCNL and RIRS). PCNL has been widely used for the treatment of upper urinary tract stones since its inception of the late 1970s[1]. However, the higher stone-free rates (SFRs) observed with this treatment are offset by the greater risk of complications associated with it[2]. To reduce complications and morbidities resulting from PCNL, micropercutaneous nephrolithotomy (Microperc) was developed[3]. The term Microperc is defined as a modified percutaneous nephrolithotomy; however, it refers to a procedure in which renal access and percutaneous nephrolithotomy are completed in one step using a 4.85-Fr all-seeing needle that allows for visualization of the entire tract during percutaneous access[4]. Retrograde intrarenal surgery (RIRS) (also termed flexible ureterorenoscopy, F-URS), is another minimally invasive measure that is an alternative to PCNL. Both of these surgeries are options for patients with small to moderate renal stones.

However, it is unclear whether Microperc is safer than and as effective as RIRS is. Several studies have examined this question[5,6,7,8,9,10,11,12,13], but the results were inconclusive. The aim of this study was to perform the first meta-analysis of research comparing Microperc with RIRS in the management of kidney stones.

## Materials and methods

A prospective study outlining the objectives, literature search strategies, inclusion and exclusion criteria, outcome measurements, and methods of statistical analysis was prepared in advance, according to the preferred reporting items for systematic reviews and meta-analysis[14].

### Search strategy

In accordance with the PRISMA guidelines[15], a systematic literature search strategy was performed by two study team members (Xiaohang Li and Jiuzhi Li) on August 21, 2017, using PubMed, Embase, and the Cochrane Library databases. The terms “‘retrograde intrarenal surgery’ or ‘RIRS’ or ‘flexible ureteroscopy’ or ‘f-URS’” and “‘micropercutaneous nephrolithotomy’ or ‘microperc’ or ‘micro’” were used as search terms. We also searched the list of references from the included studies.

### Inclusion and exclusion criteria

The selected studies were included based on the following criteria. 1) Studies that presented a comparison between Microperc and RIRS in patients with renal stones. 2) The outcome measures consisted of at least one of the following outcomes: stone-free rates, drop in hemoglobin levels, fluoroscopy time, blood transfusion status, operative time, hospitalization time and complications. Exclusion criteria included papers describing conference proceedings, repeated publications, review articles, editorials; studies of patients with musculoskeletal deformities, renal insufficiency or congenital abnormalities.

#### Study selection and data extraction

The included studies were screened and extracted by two authors (Xiaohang Li and Jiuzhi Li) independently following predefined inclusion and exclusion criteria. We contacted the authors of studies for supplement data when necessary. The first author’s name, the year of publication, baseline patient characteristics, interventions, outcome measures, statistical methods and results were used for identification purposes. The extracted outcomes were stone-free rates, drop in hemoglobin levels, fluoroscopy time, blood transfusion status, operative time, hospitalization time and complications.

### Assessment of study quality

The level of evidence (LOE) was assessed for all selected studies according to the criteria provided by the Oxford Centre for Evidence-based Medicine[16]. The methodological quality of RCTs and non-RCTs was accessed by the Cochrane Risk of Bias Tool[17] and the Newcastle-Ottawa Scale[18], respectively. This procedure was independently performed by two reviewers (Xiaohang Li and Jiuzhi Li). Any disagreements were resolved by consensus or by the adjudicating senior author (Zeng).

### Statistical analysis

All meta-analyses were performed using Review Manager Software (RevMan V.5.3, Cochrane Collaboration, Oxford, UK). Continuous data that were extracted included means and standard deviations. Pooled odds ratios (ORs) were calculated as the summary statistic for dichotomous variables. Mean differences (MDs) were calculated for continuous variables. Both ORs and MDs are reported with 95% confidence intervals (CIs). Pooled effects were determined by Z test, and statistical significance was defined as p < 0.05. The Cochrane Chi-square test and I-square test were used to assess heterogeneity among studies. A random-effects model was used for pooling when there was evidence of heterogeneity (p < 0.10, I^2^ > 50%). When there was no evidence of heterogeneity, a fixed-effects model was used. Funnel plots were used to screen for potential publication bias.

## Results

### Eligible studies and characteristics

**Fig 1:**
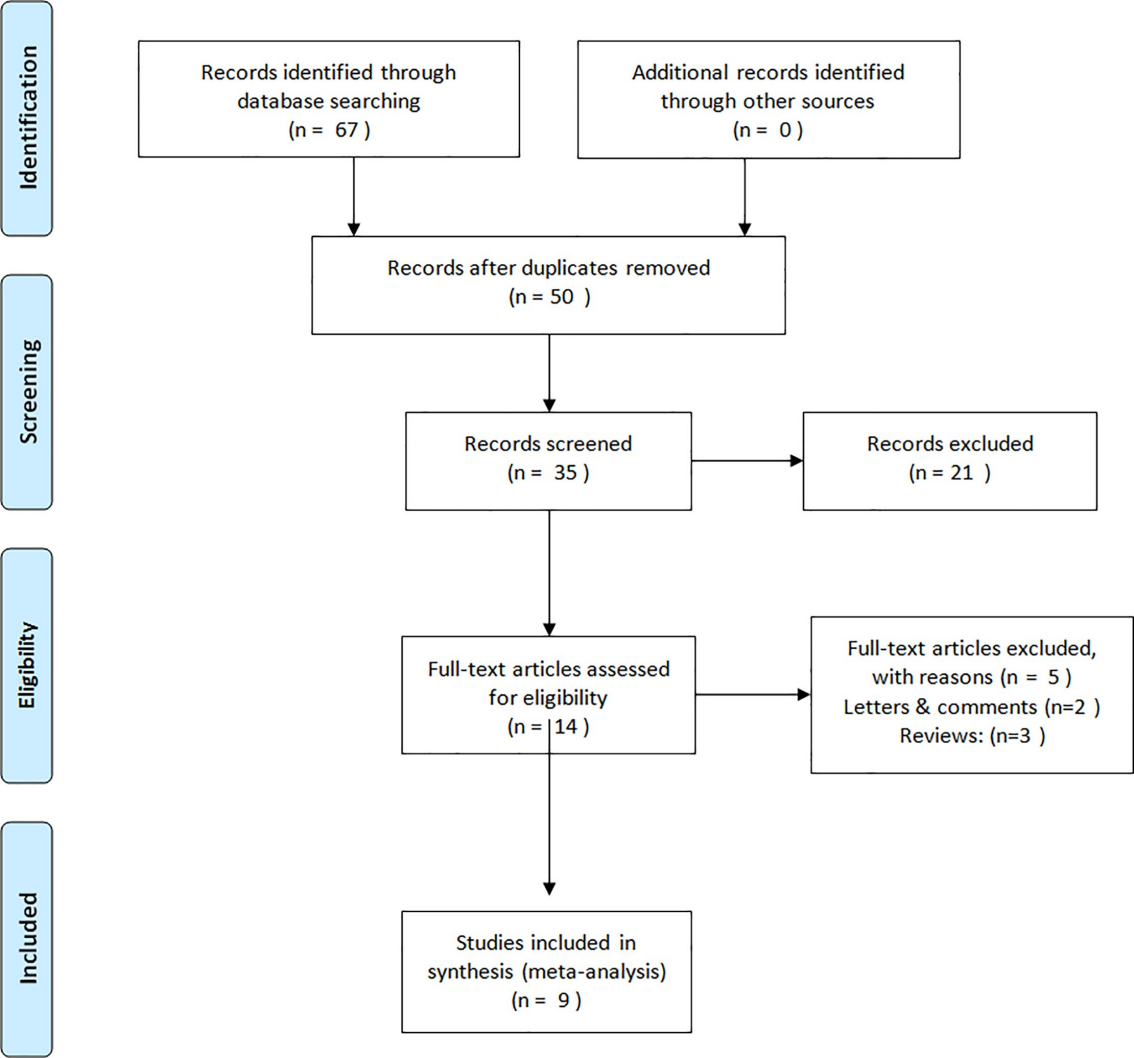
Flow diagram of studies identified, included, and excluded.

The search protocol and its results are presented in **Fig 1**. The characteristics of eligible studies are listed in **Table 1**. A total of 67 studies were identified using our search strategy. After an initial screening of titles and abstracts, 14 studies were found to meet our inclusion criteria. After further screening of the full text articles, 5 were excluded because they were reviews or editorials. No additional records were identified through the reference lists from included studies. In accordance with our predefined selection criteria, a total of nine eligible studies[5,6,7,8,9,10,11,12,13] encompassing 381 Microperc cases and 461 RIRS cases were included in our meta-analysis. Characteristics of stone size, stone location, age, gender, BMI(Body Mass Index), time point of assessing outcomes, and modalities of assessing outcomes between Microperc and RIRS were presented in **Table 2**.

**Table 1:** Microperc versus RIRS: Summary of included studies.

References	Instiution(country)	Study period	Study design	LE	Includion critera	Cases,n		Study quality
						Microperc	RIRS	
Armagan,et al[5]	Faculty of Medicine,Bezmialem Vakif university(Turkey)	2012–2014	Retrospective cohort study	3b	Renal stones <2cm, Lower pole location	68	59	7*
Bagcioglu,et al[7]	Kafkas University, Kars,(Turkey)	2013–2015	Retrospective cohort study	3b	Renal stones 1-3cm	63	48	7*
Bas,et al[6]	Department of Urology, Diskapi Yildirim Beyazid Training and Research Hospital,Ankara, Turkey	2011–2015	Retrospective cohort study	3b	Pediatric Kidney Stones,renal calculi≤2cm	45	36	8*
Cepeda,et al[8]	Servicio de Urología, Hospital Universitario Río Hortega, Valladolid, Spain	2014–2015	Retrospective cohort study	3b	Single, renal calculi≤2cm	18	17	7*
Kandemir,et al[9]	Necmettin Erbakan University(Turkey)	2013–2015	RCT	2b	Renal stones <1.5cm, single stone,lower pole location	30	30	3#
Kiremit,et al[9]	Medical Faculty of Medipol University, Istanbul,(Turkey)	2012–2014	Retrospective cohort study	3b	Renal stones 1-2cm	89	201	7*
Ramon de Fata,et al[10]	Hospital Universitario de Getafe(Madrid)	2013–2013	Retrospective cohort study	3b	Renal stones 1-3cm	8	12	5*
Sabnis,et al[11]	Muljibhai Patel Urological Hospital (India)	2011–2012	RCT	2b	Renal stones <1.5cm	35	35	5#
Sen,et al[12]	Department of Urology, School of Medicine, Gaziantep University, Gaziantep, Turkey	2015–2016	Retrospective cohort study	3b	pediatric kidney stone	25	23	7*

LE level of evidence, Microperc: micropercutaneous nephrolithotomy, RIRS: retrograde intrarenal surgery, RCT randomized controlled trials

#Using the Cochrane collaboration’s tool (score from 0 to 7)

* Using Newcastle–Ottawa Scale (score from 0 to 9), the high-quality studies were those with ≥6 stars

**Table 2:** Baseline characteristics of included studies.

References	Treatments	Stone size	Stone location	Gender(male/female)	Age(year)	BMI(SD)	Assessing SFR
Armagan et al.	Microperc	1.37±4.2 cm	Lower calyx	35:33:00	43.6±18.9	26.3±4.46	1 mo KUB/USG or CT
	RIRS	1.44±3.1 cm	Lower calyx	36:23:00	49.3±15.3	26.8±7.1	1 mo KUB/USG or CT
Bagcioglu, M et al.	Microperc	1.77±0.56 cm	NA	1.77±0.56	41.5±13.9	29.3±4.1	1 mo CT
	RIRS	1.46±0.83 cm	NA	1.46±0.83	38.5±12.6	28.4±4.3	1 mo CT
Bas,et al	Microperc	13.97±3.46 mm	NA	23:22	5.62±4.5	NA	1 mo KUB/USG
	RIRS	12.8±3.03 mm	NA	15:21	8.39±4.72	NA	1 mo KUB/USG
Cepeda et al.	Microperc	15.72±3.8 mm	NA	12:06	52.78±8.6	24.9±3.9	3 mo CT
	RIRS	16.76±3.1 mm	NA	10:07	52.41±10.51	27.8±5.7	3 mo CT
Kandemir et al.	Microperc	1.06(0.5–1.5) cm	Lower calyx	1.06(0.5–1.5)	49.7(1–78)	N/A	3 mo CT
	RIRS	1.15(0.7–1.5) cm	Lower calyx	1.15(0.7–1.5)	51.8(21–81)	N/A	3 mo CT
Kiremit et al.	Microperc	1.37±2.5 cm	NA	46:43:00	41.7±17	N/A	1 week KUB/USG or CT
	RIRS	1.415±3.7 cm	NA	111:90	41.7±17	N/A	1 week KUB/USG or CT
Ramon de Fata et al.	Microperc	1.9(0.8–3.1)cm^2^	NA	4:04	53.5(45.7–58.2)	29(23.4–35.6)	3 mo CT
	RIRS	1.3(0.6–2.6)cm^2^	NA	5:07	51(41.7–67)	28.1(24.1–30.4)	3 mo CT
Sabnis et al.	Microperc	1.10±0.23 cm^2^	NA	23:13	38.6±14.6	23.9±4.9	3 mo KUB
	RIRS	1.04±0.25 cm^2^	NA	24:11:00	43.7±12.1	24.9±4.3	3 mo KUB
Sen,et al	Microperc	12.2±2.8 mm	NA	NA	4±2.3	NA	2 Week KUB/USG
	RIRS	13.7±3.5 mm	NA	NA	10.9±3	NA	2 Week KUB/USG

SFR: stone-free rate; Microperc:micropercutaneous nephrolithotomy,RIRS: retrograde intrarenal surgery,KUB: plain film of kidney-ureter-bladder; USG: ultrasonography; CT: Computed Tomography;N/A not available

### Quality assessment of eligible studies

As shown in Table 1, LOE assessments found that two studies met Level 2 criteria and seven studies were Level 3. Following the Newcastle-Ottawa Scale, all included non-RCTs[5,6,7,8,9,11,13] with scores ≥ 7 were considered to be of high quality. The two RCTs[10,12][10,12][10,12][10,12](Sabnis and Ganesamoni et al., 2013; Kandemir and Guven et al., 2017)(Sabnis and Ganesamoni et al., 2013; Kandemir and Guven et al., 2017)(Kandemiret al. 2017,1–6,Sabniset al. 2013,355–361)Kandemir et al. (2017);Sabnis et al. (2013)[10, 12][10, 12][10, 12] assessed by the Cochrane Risk of Bias Tools were scored with five points and three points, respectively.

### Meta-analysis results in adult patients

#### SFR

**Fig 2:**
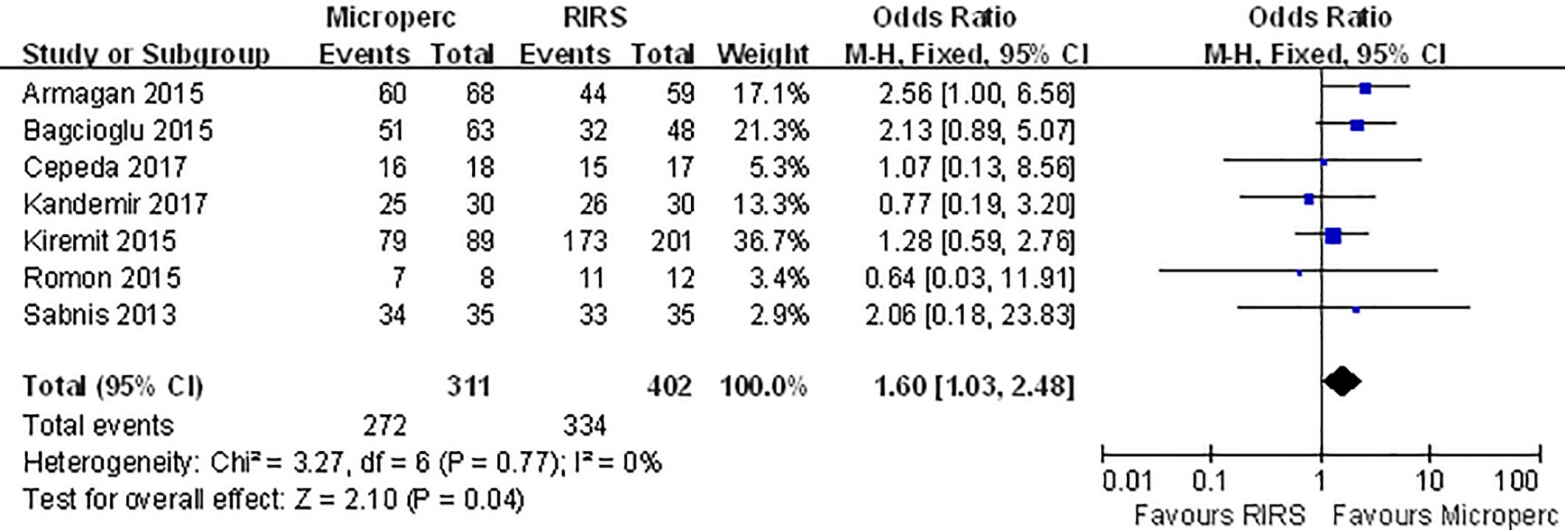
Forest plot for comparison of stone-free rate between Microperc and RIRS groups.

The efficacy of Microperc versus RIRS for renal stones in adult patients was assessed in 7 studies. Our pooled results found that Microperc was associated with a higher SFR (OR: 1.6; 95% CI, 1.03 to 2.48) compared with RIRS with low heterogeneity (I^2^ = 0%, p = 0.77) **(Fig 2).** In a subgroup analyses of SFR over a 1-month period in studies published before 2017, in studies from Turkey, and in non-RCT studies, a higher SFR was observed in the Microperc than in the RIRS group. No significant difference was found in the other subgroup analyses. **(Table 3)**

**Table 3:** Subgroup analyses for comparison of stone-free rate between Microperc and RIRS in adult patients.

Subgroups	Number of eligible studies	Heterogenerty		Combined results	
		I^2^(%)	P	OR	95%CI
≥ 1 month SFR	6	0	0.73	**1.78**	**(1.05, 3.03)**
< 1 month SFR	1	-	-	1.28	(0.89, 5.07)
Only CT for follow-up	4	0	0.6	1.46	(0.75, 2.86)
Other modalities or combined with CT for follow-up	3	0	0.53	1.7	(0.95, 3.05)
> 2017	2	0	0.8	0.85	(0.26, 2.76)
≤ 2017	5	0	0.75	**1.77**	**(1.10, 2.85)**
Turkey	4	0	0.44	**1.65**	**(1.04, 2.63)**
Other countries	3	0	0.83	1.19	(0.30, 4.70)
RCT	2	0	0.5	1	(0.30,3.34)
Non-RCT	5	0	0.71	**1.71**	**(1.07, 2.75)**

OR odds radio, CI confdence interval; The bold numbers mean the P value is < 0.05

referent surgery: RIRS

#### Hemoglobin decreases

**Fig 3:**
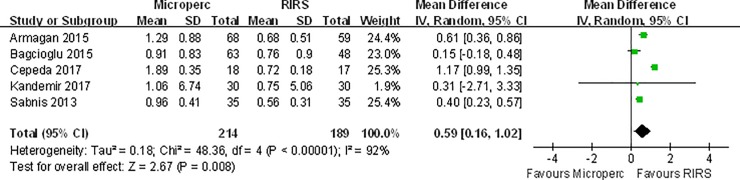
Forest plot for comparison of hemoglobin decrease between Microperc and RIRS groups.

Based on five studies, Microperc was associated with a larger decrease in hemoglobin (MD: 0.59 g/dl; 95% CI, 0.16 to 1.02) than was RIRS, with high heterogeneity observed among the trials (I^2^ = 92%, p<0.00001) **(Fig 3)**. **Table 4**presents the subgroup analyses of hemoglobin decreases between Microperc and RIRS. All results indicated that hemoglobin levels decreased significantly more with Microperc than with RIRS.

**Table 4:** Subgroup analyses for comparison of hemoglobin decrease between Microperc and RIRS in adult patients.

Subgroups	Number of eligible studies	Heterogenerty		Combined results	
		I^2^(%)	P	MD(g/l)	95%CI
RCT	2	0	0.95	**0.4**	**(0.23,0.57)**
Non-RCT	3	94	<0.00001	**0.66**	**(0.08,1.23)**
Turkey	3	59	0.09	**0.4**	**(0.00,0.79)**
Other countries	2	97	<0.00001	**0.78**	**(0.03,1.54)**
≥ 2017	2	0	0.58	**1.17**	**(0.98,1.35)**
< 2017	3	60	0.08	**0.42**	**(0.29,0.55)**

MD mean diference, CI confdence interval; The bold numbers mean the P value is < 0.05

Referent surgery: RIRS

#### Operative time

**Fig 4:**
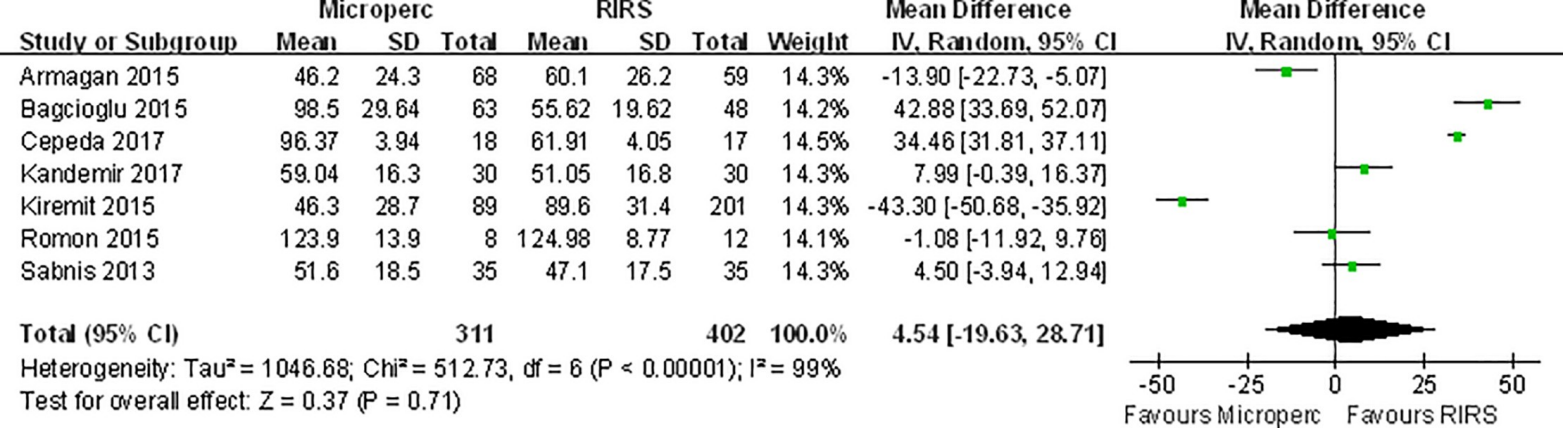
Forest plot for comparison of operative time between Microperc and RIRS groups.

Pooled data from 7 studies showed no difference between Microperc and RIRS in operative time (MD: 4.54 min; 95% CI, -19.63 to 28.71) **(Fig 4)**. Significant heterogeneity (I^2^ = 99%, P < 0.00001) existed. **Table 5**shows that the subgroup of RCT studies found that significantly more operative time was needed for Microperc compared with RIRS. Additionally, no significant difference was found in other subgroup analyses.

**Table 5:** Subgroup analyses for comparison of operative time between Microperc and RIRS in adult patients.

Subgroups	Number of eligible studies	Heterogenerty		Combined results	
		I^2^(%)	P	MD(min)	95%CI
RCT	2	0	0.57	**6.26**	**(0.31,12.2)**
Non-RCT	5	99	<0.00001	3.85	(-29.89, 37.59)
Turkey	4	99	<0.00001	1.64	(-37.67, 34.39)
Other countries	3	97	<0.00001	12.98	(-12.41,38.37)
≥ 2017	2	97	<0.00001	21.54	(-4.40, 47.47)
<2017	5	98	<0.00001	-2.24	(-31.21,26.73)

MD mean diference, CI confdence interval; The bold numbers mean the P value is < 0.05

Referent surgery: RIRS

#### Hospital stays

**Fig 5:**
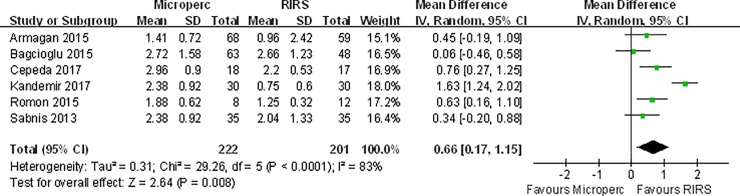
Forest plot for comparison of Hospital stay between Microperc and RIRS groups.

**Fig 5**shows a comparison of the hospital stay between the Microperc and RIRS groups. Hospital stays were longer in the Microperc group based on the pooled outcomes from 6 studies (MD: 0.66 day; 95% CI, 0.17 to 1.15), and significant heterogeneity was observed (I^2^ = 83%, P < 0.0001). The subgroup analyses by various study characteristics found that longer hospital stays were observed in the subgroups of non-RCT studies, studies performed in countries that were not Turkey, studies published before 2016, and those published after 2016 **(Table 6)**.

**Table 6:** Subgroup analyses for comparison of hospital stay between Microperc and RIRS in adult patients.

Subgroups	Number of eligible studies	Heterogenerty		Combined results	
		I^2^(%)	P	MD(day)	95%CI
RCT	2	93	0.0001	1	(-0.27,2.26)
Non-RCT	4	28	0.25	**0.5**	**(0.24, 0.76)**
Turkey	3	92	<0.00001	0.73	(-0.31, 1.77)
Other countries	3	0	0.51	**0.59**	**(0.31,0.88)**
≥ 2017	2	87	0.006	**1.21**	**(0.36, 2.06)**
< 2017	4	0	0.46	**0.38**	**(0.12,0.65)**

MD mean diference, CI confdence interval; The bold numbers mean the P value is < 0.05

Referent surgery: RIRS

#### Fluoroscopy time

**Fig 6:**
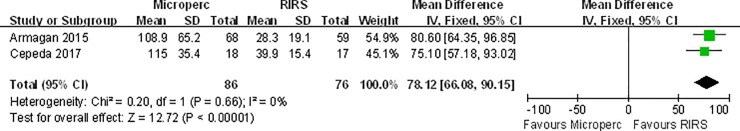
Forest plot for comparison of fluoroscopy time between Microperc and RIRS groups.

The pooled outcomes of 2 studies found that Microperc required a significantly longer fluoroscopy time (MD: 78.12 s; 95% CI, 66.08 to 90.15), and the results were not heterogeneous (I^2^ = 0%, p<0.66) **(Fig 6)**.

#### Auxiliary procedure

**Fig 7:**
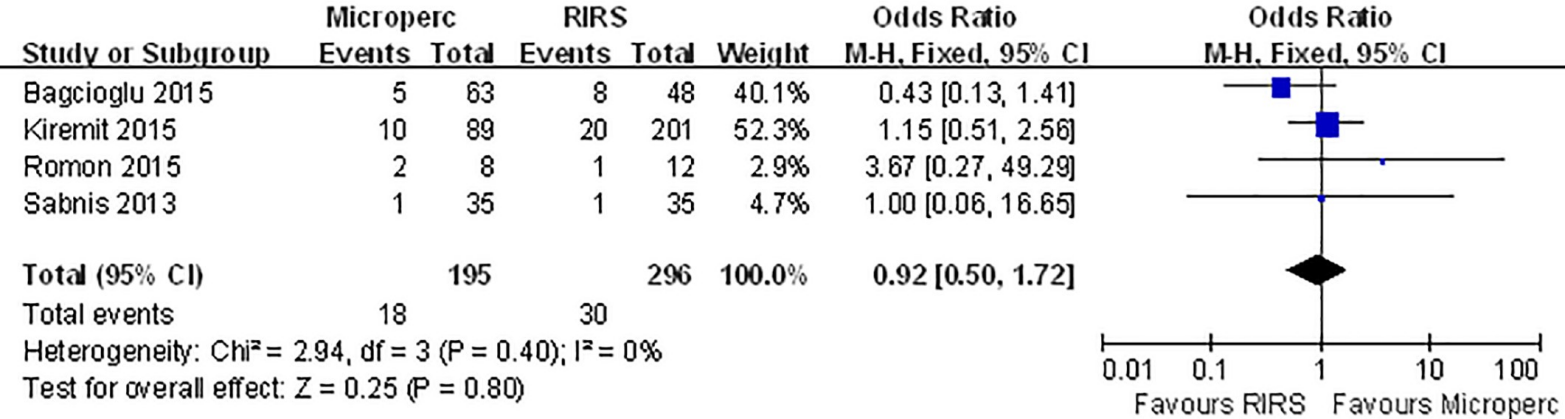
Forest plot for comparison of auxiliary procedure between Microperc and RIRS groups.

With data extracted from 4 studies, our meta-analysis showed no difference between the groups regarding auxiliary procedure rate (OR: 0.92; 95% CI, 0.50–1.72) **(Fig 7),** with little evidence of heterogeneity of the effects (I^2^ = 0%, p = 0.4). In the subgroup analyses, no groups indicated a significant difference in the auxiliary procedure **(Table 7)**.

**Table 7:** Subgroup analyses for comparison of auxiliary procedure between Microperc and RIRS in adult patients.

Subgroups	Number of eligible studies	Heterogenerty		Combined results	
		I^2^(%)	P	OR	95%CI
RCT	1	-	-	1	(0.06,16.65)
Non-RCT	3	32	0.23	0.91	(0.38, 2.21)
Turkey	2	44	0.18	0.84	(0.43, 1.63)
Other countries	2	0	0.51	2.02	(0.31,12.95)
≥ 2015	3	32	0.23	0.92	(0.49,1.74)
< 2015	1	-	-	1	(0.06,46.65)

OR odds radio, CI confdence interval, NA not applicable

referent surgery: RIRS

#### Complication rate

**Fig 8:**
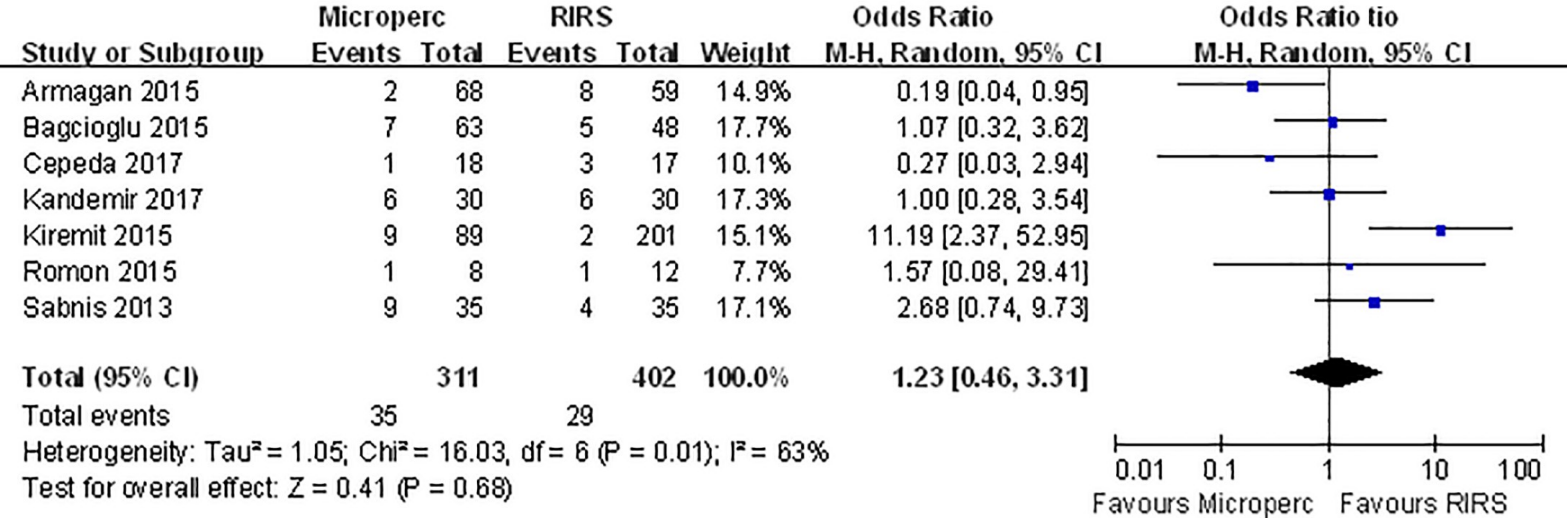
Forest plot for comparison of total complication between Microperc and RIRS groups.

**Fig 9:**
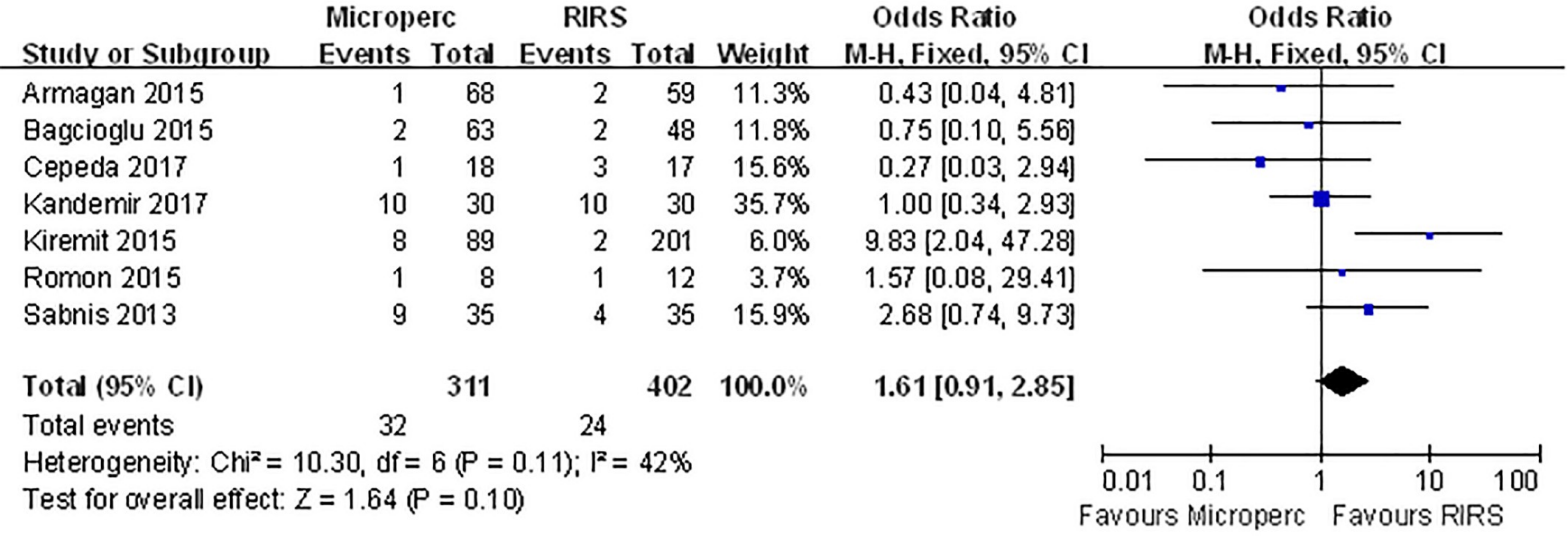
Forest plot for comparison of complication 1–2 between Microperc and RIRS groups.

**Fig 10:**
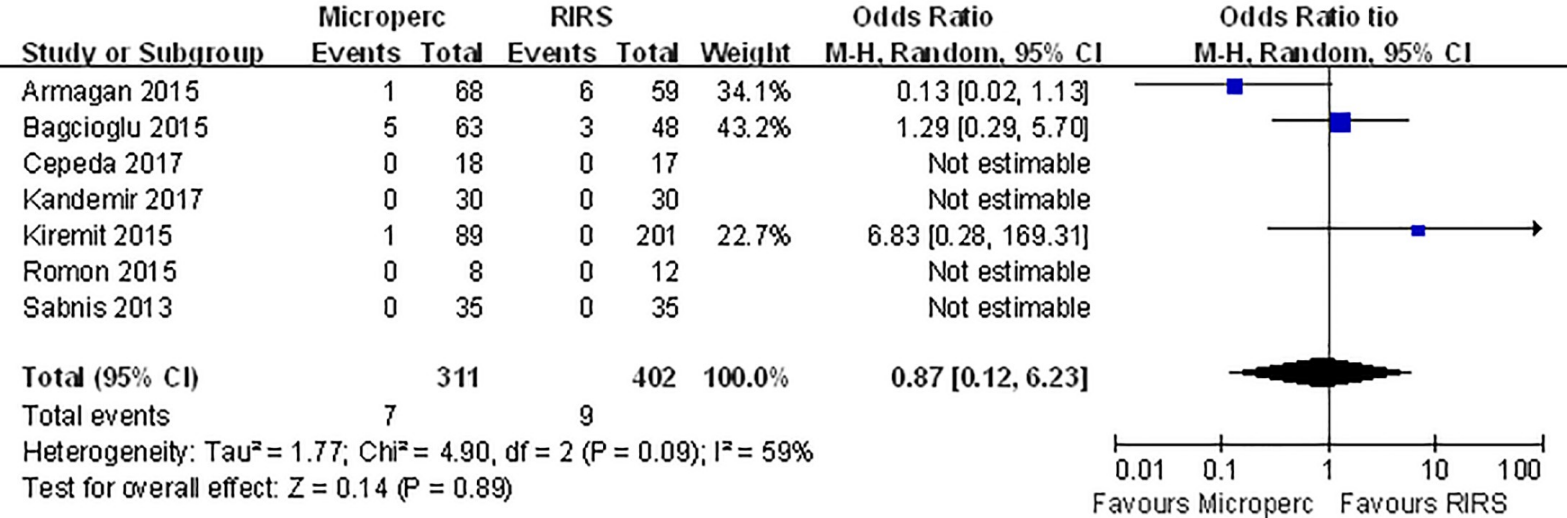
Forest plot for comparison of complication 3–4 between Microperc and RIRS groups.

The overall complication rate was compared in 7 studies. The meta-analysis showed no difference between the two groups (OR: 1.23; 95% CI, 0.46–3.31) and significant heterogeneity (I^2^ = 63%, P = 0.01) was observed **(Fig 8)**. When we classified complications using the modified Clavien system, no significant difference was found between the two groups in minor (Clavien I or II) complication rates **(Fig 9)** or major (Clavien III–V) complication rates **(Fig 10)**. In subgroup analyses, no subgroups indicated a significant difference in total complication rate **(Table 8)**.

**Table 8:** Subgroup analyses for comparison of total complication rate between Microperc and RIRS in adult patients.

Subgroups	Number of eligible studies	Heterogenerty		Combined results	
		I^2^(%)	P	OR	95%CI
RCT	2	13	0.28	1.64	(0.74, 3.98)
Non-RCT	5	72	0.006	1.04	(0.23, 4.77)
Turkey	4	77	0.004	1.23	(0.29, 5.28)
Other countries	3	27	0.25	1.5	(0.56,3.99)
≥ 2017	2	0	0.34	0.73	(0.25, 2.15)
< 2017	5	71	0.008	1.59	(0.43,5.92)

OR odds radio, CI confdence interval

referent surgery: RIRS

### Meta-analysis results in pediatric patients

The efficacy of Microperc versus RIRS for renal stones in pediatric patients was assessed in 2 studies. The pooled results comparing Microperc and RIRS in pediatric patients are provided in Table 9. Only one study compared fluoroscopy time between Microperc and RIRS, and it found that Microperc had a higher fluoroscopy time than did RIRS. In addition, no significant differences were found in operation time, SFR, hospital stay, total complication, minor complication rates (Clavien I or II) or major complication rates (Clavien III–V).

**Table 9:** Pooled outcomes for comparison between Microperc and RIRS in pediatric patients.

Outcome	Number of eligible studies	Heterogenerty		Combined results	
		I^2^(%)	P	MD/OR	95%CI
Operation time	2	55	0.14	7.62 min	(-1.03, 16.27)
Fluoroscopy time	1	-	-	**35.14 min**	**(20.91, 49.37)**
SFR	2	0	0.58	0.79	(0.31,2.01)
Hospital stay	2	0.0004	92	0.31 day	(-0.51, 1.13)
Total complication	2	0.97	0	0.72	(0.27, 1.92)
Complication 1–2	2	0.6	0	0.6	(0.24, 1.51)
Complication 3–4	2	0.65	0	0.5	(0.06, 3.95)

MD mean diference, OR odds radio, CI confdence interval; The bold numbers mean the P value is < 0.05

Referent surgery: RIRS

### Publication bias

An inverted funnel plot was used to assess publication bias. No bias was detected for the results presented in this meta-analysis.

## Discussion

To the best of our knowledge, this is the first systematic review and meta-analysis comparing the efficacy and safety of Microperc and RIRS for treating renal stones. This analysis was based on a total of nine studies including 842 patients. Seven studies were conducted in adult patients, while 2 studies were conducted in pediatric patients. Our results demonstrated that adult patients receiving Microperc had a significantly higher SFR, longer hospital stay, longer fluoroscopy time, and larger decline in hemoglobin levels. Furthermore, no significant differences were found in relation to operative time, stone-free rate, complication rate or auxiliary procedures. Therefore, Microperc may be considered an effective and safe surgical intervention for patients with renal stones and perhaps might even be more effective than RIRS is. However, longer hospital stays, longer fluoroscopy time, and larger declines in hemoglobin should be carefully considered.

RIRS is the established gold standard of surgical correction for moderate renal stones (<20 mm) and has demonstrated a good SFR and a small risk of major complications[19]. Our results showed that the SFR of Microperc was slightly higher than that of RIRS. Furthermore, adult patients treated by Microperc had a higher average stone-free rate (87.5%) than did adult patients treated by RIRS (83.1%). Some studies [20,21,22] also reported SFRs ranging from 90% to 93% in adult patients with renal stones treated by Microperc.

Imaging modality (KUB in one study, CT in four studies, and KUB/USG or CT in one study) and the time at which SFR was assessed (1 week in 1 study, 1 month in 2 studies, 3 months in 4 studies) varied among the included studies **(Table 2)**. In the subgroup analyses of SFR over 1 month, a higher SFR was found in the Microperc group than in the RIRS group. No significant difference was found in the subgroup analyses of SFR below 1 month or within any imaging modality **(Table 3)**. Besides, no significant difference was found in RCT studies of subgroup analyses. In fact, RCT is the gold standard for the evaluation of interventions. However, Microperc is very different to RIRS in operative treatment. Therefore, it is hard to design a good RCT comparing the safety and efficacy between Microperc and RIRS because of impossibility of allocation concealment and blinding. Besides, we found that the reporting bias of Kandemir[10]’s RCT was high because of selective reporting. Thus, the evidence found the subgroup analyses of 2 RCTs may be biased. In order to confirm the stability of pooled results, a sensitive analysis was conducted to interpret the results of SFR. After omitting an observation study[9] with low-quality and/or an RCT[10] with high-bias, Microperc was also associated with a higher SFR compared with RIRS. Therefore, the summary of risk estimates was relative stable.

With respect to total complications, no significant difference was reported in 6 included studies. Our results also demonstrated that no significant difference was found between Microperc and RIRS groups. In addition, no significant difference in mild complication rate (Clavien I or II) or severe complication rate (Clavien III–V) existed between the Microperc and RIRS procedures. In the subgroup analyses, none of the subgroups indicated a significant difference in total complication rate. As shown in **S1 Table**, we also discovered that the overall rate of bleeding-related complications, including hemorrhage (Microperc 1.6%; RIRS 0), blood transfusion (Microperc 1.3%; RIRS 0) and A-V fistula (Microperc 0.3%; RIRS 0), was higher for Microperc group compared to RIRS group. On the other hand, infection-related complications were more common in the RIRS group than in the Microperc group, with respect to fever (Microperc 2.9%; RIRS 3.2%) and urinary tract infection (Microperc 0.6%; RIRS 1%).

The pooled results of 5 eligible studies showed that larger declines in hemoglobin levels were identified for Microperc than RIRS. Although the heterogeneity was high, all results from subgroup analysis indicated that hemoglobin levels decreased significantly more to Microperc than with RIRS. Armagan, et al.[5] pointed out that some hemorrhage may occur during Microperc because the procedure features puncture.

In this analysis, Microperc resulted in longer hospital stays than RIRS. A sensitivity analysis was performed to assess the effect of one single study on the pooled estimate by sequentially excluding each study in one turn. No studies were found to be significantly influencing the summary of risk estimate. The most likely reason that a longer time of hospital stays was found in Microperc might be the higher bleeding-related complication rate for Microperc than RIRS. Patients undergoing RIRS surgery could return to society faster than those undergoing Microperc surgery.

Only two studies reported data comparing fluoroscopy time in adult patients. Each of the two studies revealed a longer fluoroscopy time in the Microperc group than in the RIRS group. Only Kandemir, et al[10] presented the definition of fluoroscopy time: the sum of instant and short time views during the process which was specified on the Ziehm Vision R C-Arm System panel (Germany). Our pooled result also demonstrated that a longer fluoroscopy time was needed for Microperc compared with RIRS. Due to high recurrence rate of renal stones, the difference in fluoroscopy time could be translated into clinical significance by considering the benefit to patients. In addition, RIRS should be considered for adult patients for whom radiation exposure should be minimized.

Pooled analysis of seven studies revealed no significant difference between Microperc and RIRS in operative time among adult patients. Only 2 studies[5,6] presented the definition of operative time. Operative time was defined as the duration of time from the beginning of renal puncture to the removal of the percutaneous system. Operative time was nearly relative with surgical experience and surgical technique. The variations among studies during the Microperc procedures were shown in guidance modalities, the size of the open-end ureteral catheter, the size of the working channel and the placement of postoperative JJ stents (S2 Table). RIRS also had variations in dilation of ureter orifice, ureteral access sheath, size of flexible ureterorenoscope, the use of a basket. And he placement of postoperative JJ stents (S3 Table). All these variations above can contribute to the heterogeneity of results.

No significant difference in auxiliary procedure was found between Microperc and RIRS in adult patients. The auxiliary procedure rate was 2.86%-25% in Microperc and 2.86%-16.67% in RIRS[6,9,11,12]. In our study, removal of JJ stents after the main surgery was not considered as an auxiliary procedure because the criteria varied among the studies with regard to the use of JJ stents (S2 and S3 Tables).

Only 2 studies reported the comparison between Microperc and RIRS in pediatric patients. Limited evidence of pooled results showed that no significant difference was found in operation time, SFR, hospital stays, total complications, minor complication rates (Clavien I or II) or major complication rates (Clavien III–V). More studies of pediatric patients are needed (Table 9).

There are some limitations in our study. Above all, seven included studies were non-randomized, and only two RCTS were available for inclusion. Although the overall quality of the studies was acceptable, there were some potential risk of biases originating from the eligible studies. Although subgroup and sensitivity analysis were conducted to confirm the stability of risk estimate. Potential bias could be existed, so further high-quality, multi-center RCTs are necessary to confirm our results. In addition, heterogeneity among studies was found to be high for several parameters, including operative time, fluoroscopy time, hemoglobin decrease, and hospital stay. This heterogeneity can be explained by differences in surgical technique and experience and in the outcome definitions among the studies. Finally, the limited number of studies and relatively small number of patients included in the present analysis might not achieve sufficient power to obtain valid results.

## Conclusion

Our meta-analyses demonstrated that the adult patients receiving Microperc had a higher SFR, significantly longer hospital stay, longer fluoroscopy time, and a larger decrease in hemoglobin levels. No significant differences were found in operative time, complication rate or auxiliary procedures between Microperc and RIRS. As a result, compared with RIRS, Microperc might be more effective than RIRS is in adult patients. However, longer hospital stay, longer fluoroscopy time and a greater decline in hemoglobin levels should be carefully considered. Finally, further high-quality multicenter RCTs are still needed to confirm our results.

## Supporting information

S1 File PRISMA 2009 checklist. (DOC)

S1 Table Comparison of complications between Microperc and RIRS. (DOCX)

S2 Table Variations in Microperc techniques. (DOCX)

S3 Table Variations in RIRS techniques. (DOCX)

## Abbreviations

Microperc: micropercutaneous nephrolithotomy
RIRS: retrograde intrarenal surgery
CT: Computed Tomography
KUB: plain film of kidney-ureter-bladder
USG: ultrasonography
CI: confidence interval
MD: Mean difference
OR: odds ratio
