# Supplementary material for: Micropercutaneous nephrolithotomy versus retrograde intrarenal surgery in the treatment of renal stones: A systematic review and meta-analysis
Source: PLoS One. 2018 Oct 19;13(10):e0206048. doi: 10.1371/journal.pone.0206048 (PMC6195289; doi:10.1371/journal.pone.0206048)
Supplement: S1 Table — (DOCX) [file pone.0206048.s002.docx]

| S1 Table Comparison of complications between Microperc and RIRS | | | | | | | | | | | | | | | | | | |
| --- | --- | --- | --- | --- | --- | --- | --- | --- | --- | --- | --- | --- | --- | --- | --- | --- | --- | --- |
|  | Fever | | Hemorrhage | | Ureteral mucosal injury | | Renal colic | | Urinary tract infection | | Pelvic perforation | | Blood transfusion | | Steinstrasse | | A-V fistula | |
|  | Microperc | RIRS | Microperc | RIRS | Microperc | RIRS | Microperc | RIRS | Microperc | RIRS | Microperc | RIRS | Microperc | RIRS | Microperc | RIRS | Microperc | RIRS |
| Armagan et al. |  |  |  |  |  |  |  |  | 1(1.5%) | 2(3.4%) |  |  |  |  | 1(1.5%) | 6(10.2%) |  |  |
| Bagcioglu et al. |  |  |  |  |  | 1(2.1%) | 2(3.2%) | 1(2.1%) |  |  |  |  |  |  | 5(7.9%) | 3(6.3%) |  |  |
| Cepeda et al. | 1(5.6%) | 2(11.8%) |  |  |  | 1(5.9%) |  |  |  |  |  |  |  |  |  |  |  |  |
| Kandemir et al. | 4(13.3%) | 4(13.3%) |  |  |  |  | 4(13.3%) | 4(13.3%) | 1(3.3%) | 2(6.7%) |  |  | 1(3.3%) |  |  |  |  |  |
| Kiremit et al. | 1(1.1%) | 2(1%) |  |  |  |  | 4(4.5%) |  |  |  |  |  | 3(3.4%) |  |  |  | 1(1.1%) |  |
| Ramón et al. |  | 1(8.3%) |  |  |  |  |  |  |  |  |  |  |  |  |  |  |  |  |
| Sabnis et al. | 3(8.6%) | 4(11.4%) | 5(14.3%) |  |  |  |  |  |  |  | 1(2.9%) |  |  |  |  |  |  |  |
| Overall | 9(2.9%) | 13(3.2%) | 5(1.6%) | 0(0%) | 0(0%) | 2(0.5%) | 10(3.2%) | 5(1.2%) | 2(0.6%) | 4(1%) | 1(0.3%) | 0(0%) | 4(1.3%) | 0(0%) | 6(1.9%) | 9(2.2%) | 1(0.3%) | 0(0%) |
