# Supplementary material for: Micropercutaneous nephrolithotomy versus retrograde intrarenal surgery in the treatment of renal stones: A systematic review and meta-analysis
Source: PLoS One. 2018 Oct 19;13(10):e0206048. doi: 10.1371/journal.pone.0206048 (PMC6195289; doi:10.1371/journal.pone.0206048)
Supplement: S2 Table — (DOCX) [file pone.0206048.s003.docx]

| **S2 Table** Variations in Microperc techniques | | | | |
| --- | --- | --- | --- | --- |
|  | Guidance | Open-end ureteral catheter | working channel | Postop stents |
| Armagan et al. | Fluoroscopic | R 6/7 Fr | 4.85 F needle | NA |
| Bagcioglu et al. | Fluoroscopic | R 6-Fr | 4.85 F needle | S |
| Cepeda et al. | Fluoroscopic | R 8 F | 4.85 F needle | R |
| Kandemir et al. | Fluoroscopic/Ultrasonographic | R NA | 4.85 F needle | NA |
| Kiremit et al. | Fluoroscopic/Ultrasonographic | R NA | 4.85 F needle | S |
| Ramón et al. | Fluoroscopic/Ultrasonographic | R 8 F | 4.85 F needleF/4.85 F needle with 8 F sheath | R |
| Sabnis et al. | Fluoroscopic/Ultrasonographic | R 7F | 4.85 F needle | S |
| R routine use, S selective use | | | | |
