# Supplementary material for: Micropercutaneous nephrolithotomy versus retrograde intrarenal surgery in the treatment of renal stones: A systematic review and meta-analysis
Source: PLoS One. 2018 Oct 19;13(10):e0206048. doi: 10.1371/journal.pone.0206048 (PMC6195289; doi:10.1371/journal.pone.0206048)
Supplement: S3 Table — (DOCX) [file pone.0206048.s004.docx]

| **S3 Table** Variations in RIRS techniques | | | | | |
| --- | --- | --- | --- | --- | --- |
|  | Dilation of ureter orifice | Ureteral access theath | Size of flexible ureterorenoscope | Basket | Postop JJ stents |
| Armagan et al. | NA | R | 7 F | S | R |
| Bagcioglu et al. | Ureteroscopy | R 11/13-Fr | NA | S | S |
| Cepeda et al. | NA | R Less than 14 Fr | NA | R | R |
| Kandemir et al. | NA | R | NA | S | S |
| Kiremit et al. | Ureteroscopy/Balloon dilators | R | NA | No | S |
| Ramón et al. | Ureteroscopy | R 11-14 F | NA | R | R |
| Sabnis et al. | Fascial dilators | R 12 F | 7.5 F | S | S |
| R routine use, S selective use | | | | | |
